# Archetypal Analysis Reveals Consistent Visual Field Patterns for Stimulus Sizes III and V in Glaucoma and NAION

**DOI:** 10.1167/tvst.13.12.15

**Published:** 2024-12-12

**Authors:** David Szanto, Michael Wall, Luke X. Chong, Brian Woods, Tobias Elze, Jui-Kai Wang, Mona Garvin, Randy Kardon, Mark J. Kupersmith

**Affiliations:** 1 Department of Neurology, Icahn School of Medicine at Mount Sinai, New York, NY, USA; 2 Department of Ophthalmology and Visual Sciences, University of Iowa, Iowa City, IA, USA; 3 School of Medicine, Deakin University, Geelong, Australia; 4 Irish Clinical Academic Training Programme, Department of Ophthalmology, Galway University Hospital, Galway, Ireland, UK; 5 Schepens Eye Research Institute, Harvard Medical School, Boston, MA, USA; 6 Center for the Prevention and Treatment of Visual Loss, Iowa City VA Health System, Iowa City, IA, USA; 7 Department of Ophthalmology and Visual Sciences, University of Iowa, Iowa City, IA, USA; 8 Department of Electrical and Computer Engineering, University of Iowa, Iowa City, IA, USA; 9 Departments of Neurology, Ophthalmology and Neurosurgery, CNIIC, Icahn School of Medicine at Mount Sinai, NY, USA

**Keywords:** archetypal analysis, glaucoma, NAION

## Abstract

**Purpose:**

Disorders of the anterior optic nerve cause quantifiable patterns, or archetypes (AT), in visual fields (VFs) obtained using standardized automated perimetry using stimulus size III (size III). VFs with stimulus size V (size V) can reduce retest variability in eyes with moderate to severe loss. We postulated that VF testing using both stimuli would show similar ATs in eyes with glaucoma and nonarteritic anterior ischemic optic neuropathy (NAION).

**Methods:**

We used 1969 same-day pairs of 24-2 size III and size V VFs from two datasets. QRK207 is the largest NAION study to date, and the VIPII study measured same-day VFs across many stimulus sizes. We censored raw sensitivities of less than 21 dB for size III and 24 dB for size V and age-standardized to total deviations, before archetypal analysis (AA). We compared the ATs between the two stimuli and the combined data.

**Results:**

Using 14 ATs for both glaucoma and NAION, AA captured similar patterns between the two stimuli in both diseases with 87% of AT pairings having a cosine similarity of 0.8 or greater. The combined ATs retained the patterns in the separate stimuli VFs.

**Conclusions:**

AA shows that size V VFs provide quantifiable patterns of loss similar to size III. This aids in comparing stimulus sizes for monitoring VF patterns in disease progression.

**Translational Relevance:**

AA shows similar quantifiable patterns of VF loss with size III or size V, supporting the use of size V to monitor eyes with moderate to severe VF loss.

## Introduction

Disorders of the anterior optic nerve cause visual loss and standard automated perimetry testing of the visual field (VF) is an important method used in diagnosis and monitoring. Standard automated perimetry measures visual sensitivities at multiple test locations to detect deficits most commonly in the central 30° of vision. Goldmann stimulus size III (size III) is used commonly, but has limitations in eyes with moderate to severe deficits owing to its narrow useful dynamic range (4 mm^2^, 0.43°).^1^ In contrast, stimulus size V (size V), which uses a larger stimulus, offers a wider useful dynamic range (64 mm^2^, 1.72°) and is more reliable and repeatable for testing eyes with advanced VF loss.^2^ Size V is now used by some clinicians to assess severely depressed VFs.^3^ Even with the use of size V, testing points with low sensitivities eventually leads to variable and unreliable results. Censoring is done when the probability of a value being missing is equal to the probability of that value being below a detection limit. The signal-to-noise ratio decreases dramatically in standard automated perimetry below 25 dB.^4^ This allows for censoring sensitivities below this level. It has been shown that this type of censoring can improve the repeatability of testing results and this can be done for both nonarteritic anterior ischemic optic neuropathy (NAION) and glaucoma.^5^ Glaucoma and NAION are leading causes of vision loss in adults. Both conditions lead to irreversible visual impairment, ranging from mild VF defects to blindness, significantly diminishing quality of life, and are monitored with VF testing.^6^^,^^7^ The VF loss for both often show patterns typical of anterior optic nerve head injury. These patterns of VF loss can be quantified using archetypal analysis (AA), a dimensionality reduction technique for unsupervised analysis.^8^ This method identifies extremal points within a dataset, allowing each data point to be represented as a combination of these archetypes (ATs).^8^ AA demonstrates regional VF deficits in eyes with papilledema, optic neuritis, and glaucoma.^9^^–^^11^

We hypothesized that VFs obtained using size III and size V would show similar patterns in ATs for glaucoma and NAION. Additionally, we explored whether censoring and age standardization for size III and size V VFs,^5^ using VFs obtained with both stimuli, would also demonstrate similar ATs. If successful, clinicians and investigators could then use methods like AA for VFs where the stimulus size is optimized to reduce variability.

## Methods

This study was approved by the Institutional Review Board of the Icahn School of Medicine at Mount Sinai and the University of Iowa Institutional Review Board and required no additional consent as the data used were deidentified and from participants who had consented for use of their data at multiple study institutions. The study was conducted according to the tenets of the Declaration of Helsinki.

### Glaucoma

VF data using stimulus sizes III and V for glaucoma were obtained from the VIPII study investigating differences in variability between different perimetric stimuli sizes and their ability to distinguish between healthy and damaged VFs in glaucoma patients.^12^^,^^13^ The study compared abnormal test locations across various stimuli sizes. This research involved previously reported data from 120 participants with moderate to severe VF loss owing to glaucoma, who underwent same-day VF testing using both 24-2 Swedish Interactive Threshold Algorithm standard size III and full-threshold size V at the University of Iowa Department of Ophthalmology and Visual Sciences. Participants were included if they had glaucomatous optic disc changes with abnormal conventional automated perimetry and were diagnosed with primary, secondary, or normal tension glaucoma, without any other vision-affecting diseases. Exclusion criteria were cataract causing visual acuity worse than 20/30, pupil size less than 2.5 mm, age less than 19 years, or pregnancy at the time of study entry.

### NAION

NAION data were obtained from the Quark207 trial, a multinational, prospective, randomized controlled trial designed to investigate the safety and efficacy of a biologic in individuals aged 50 to 80 years who were diagnosed with NAION within 14 days of vision loss and met study entry criteria. The study included 729 participants who were tested using both the 24-2 Swedish Interactive Threshold Algorithm standard size III and full threshold size V, which was added after recruitment began. Both stimuli tests were performed on the same day.

### Data Censoring and Conversion

Raw sensitivity values were recorded for each test. For participants with glaucoma, participants had same-day VFs every 6 months for 4 years. For participants with NAION, same-day VFs using both stimuli were recorded for participants at month 6 (*n* = 482), month 12 (*n* = 405), and varying unscheduled times (*n* = 29). Prior work showed that the optimal censoring threshold to equate sensitivities for moderate to severe glaucoma and NAION are 21 dB for size III and 24 dB for size V.^5^ We censored sensitivities by replacing values below the defined thresholds with that value. Then, we age-standardized sensitivities and converted them to total deviation (TD) values using a normative database for size III and derived from VF data from 60 healthy patients tested twice for size V.^4^ We excluded 26 VFs with a false-positive error rate of greater than 20% to prevent artificially elevated positive TDs, as AA generates patterns from the edges of the data space, which could lead to misleading results

### Data Visualization and Analysis

We performed all statistical analyses using Python 3.8.8. To run AA, we used the “archetypes” module within the Python programming environment.^14^ We calculated the residual sum of squares (RSSs) for our VF data and the model's reconstruction of the data, using between two and 20 ATs. For each AA model, we used a 10-fold cross-validation to select the optimal number of ATs. This involved dividing the data into 10 subsets, where each subset was used as the testing set once while the remaining subsets served as the training set. We generated plots for each disease by size III to display the relationship between the number of ATs and their corresponding RSS values for each disease. We chose the number of ATs by applying the elbow method to identify a range of feasible options, and then balanced selecting a lower number of ATs with achieving an RSS score close to the global minimum. We normalized the sum of the relative weights (RWs) of all ATs to equal 100%. The ATs were ordered by RW, representing their frequency within the dataset. Each VF was decomposed into component ATs such that it was represented by a set of AT weighting coefficients that summed to 100%.

To group similar ATs by disease, we used cosine similarity, a metric that quantifies the similarity between two VFs, and a recognized method in VF analysis,^15^ by calculating the normalized dot product of their values by treating each VF as a 52-point vector. Cosine similarity generates scores ranging from −1 to 1, where values closest to 1 indicate a high degree of similarity between patterns. Importantly, cosine similarity is independent of intensity; if the TD values at each location were doubled, the similarity score would remain unchanged. This property is particularly relevant because ATs are the weighted sums of patterns. This strategy allowed us to compare the shape of the disease-related VF defects without confounding the analysis with differences in severity.

We calculated the cosine similarity between every possible AT pair between size III and size V VFs. Starting from the pairs with the highest cosine similarity scores, we sequentially grouped the most similar ATs. Once an AT was assigned to a pair, it was excluded from further pairings to ensure that each AT was used only once.

We then ran AA on both stimuli to create a third AT map, and decomposed VFs from both stimuli to RW. We compared AT RWs with a Wilcoxon signed-rank test at a significance level of α = 0.05.

## Results

We analyzed a total of 1969 VFs from one eye of 698 participants, of which 120 had moderate to severe glaucoma and 578 had NAION. Among participants with glaucoma, the mean age was 67.8 ± 9.3 years with 39% being male, whereas for those with NAION, the mean age was 61.2 ± 7.7 years where 69% were male (Table 1). The mean TD after censoring for participants with glaucoma was −4.7 ± 3.7 dB for size III and −4.2 ± 3.9 dB for size V, where 35% and 22% of sensitivities were censored, respectively. For participants with NAION, the mean TD was −7.2 ± 3.3 dB for size III and −7.4 ± 3.8 dB for size V where 63% and 52% of sensitivities were censored, respectively. Participants with glaucoma had an uncensored mean TD of −9.5 dB for size III and −7.1 dB for size V. Those with NAION had an uncensored mean TD of −17.3 dB for size III and −15.4 dB for size V.

**Table 1.** Study Participant Characteristics: Demographics of Participants With Glaucoma and NAION

	# VF Pairs	Age (Years)	% Male	% Right Eye
Glaucoma (*n* = 120)	1053	67.8 ± 9.3	39	53
NAION (*n* = 578)	916	61.2 ± 7.7	69.3	51

**Figure 1.**
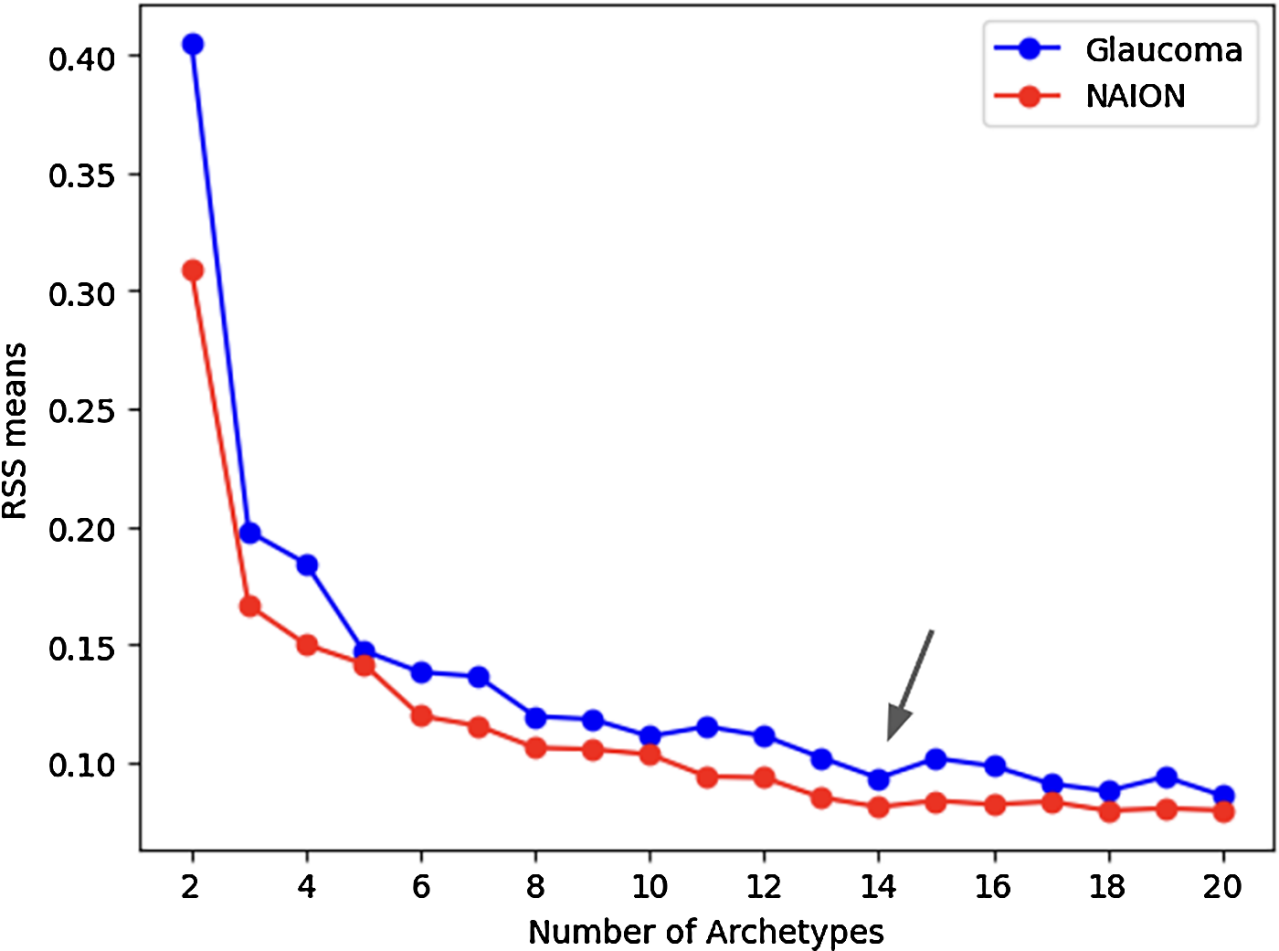
RSSs by disease. Plot of RSSs against number of ATs to determine the best number of ATs for VFs using stimulus size III in glaucoma and NAION. The number of ATs used was 14 for both diseases, at which point the curve flattens, balancing predictive accuracy and overfitting.

**Figure 2.**
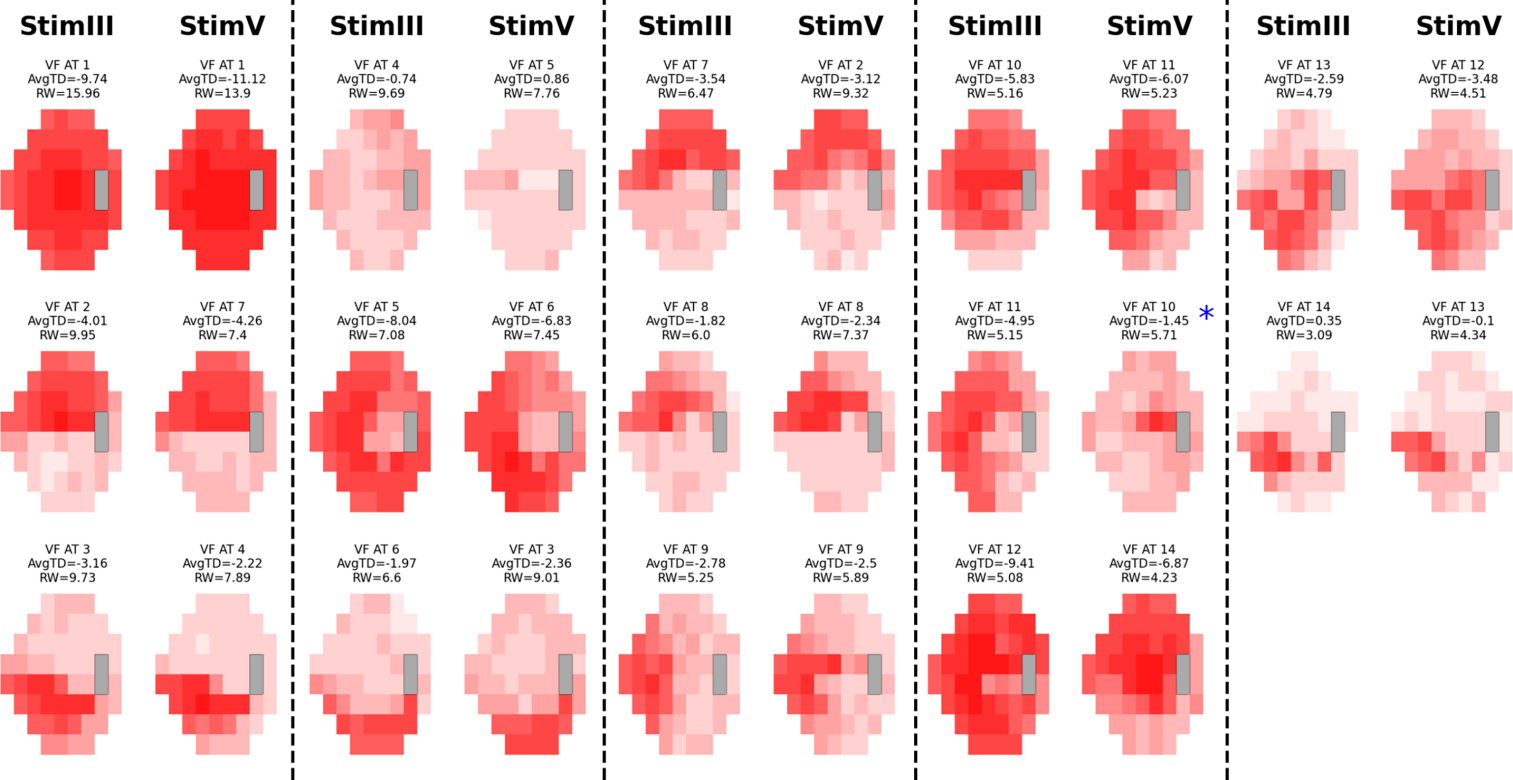
Glaucoma AT stimulus comparison map. VF patterns in glaucoma using stimulus sizes III and V, displayed side by side. The different shades of red within each AT represent TD values. Size III patterns are on the left, with the most closely matching size V patterns on the right. There is a near 1:1 pattern similarity for corresponding ATs except between size III AT12 and size V AT5.

**Figure 3.**
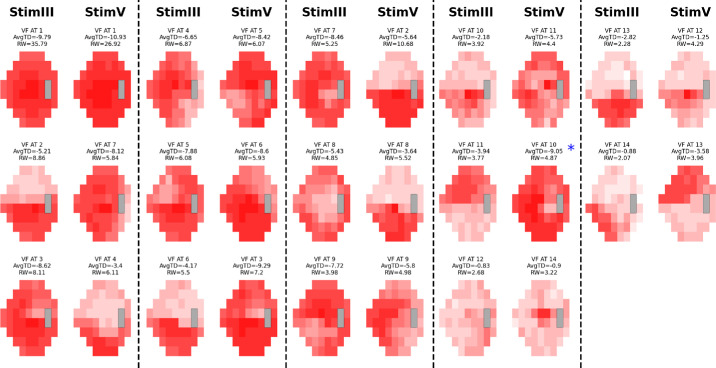
NAION AT stimulus comparison map. VF patterns in NAION using stimulus sizes III and V, displayed side by side. The different shades of red within each AT represent TD values. Size III patterns are on the left, with the most closely matching size V patterns on the right. There is a near 1:1 pattern similarity for corresponding ATs except between size III AT12 and size V AT14 and size III AT14 and size V AT9.

**Figure 4.**
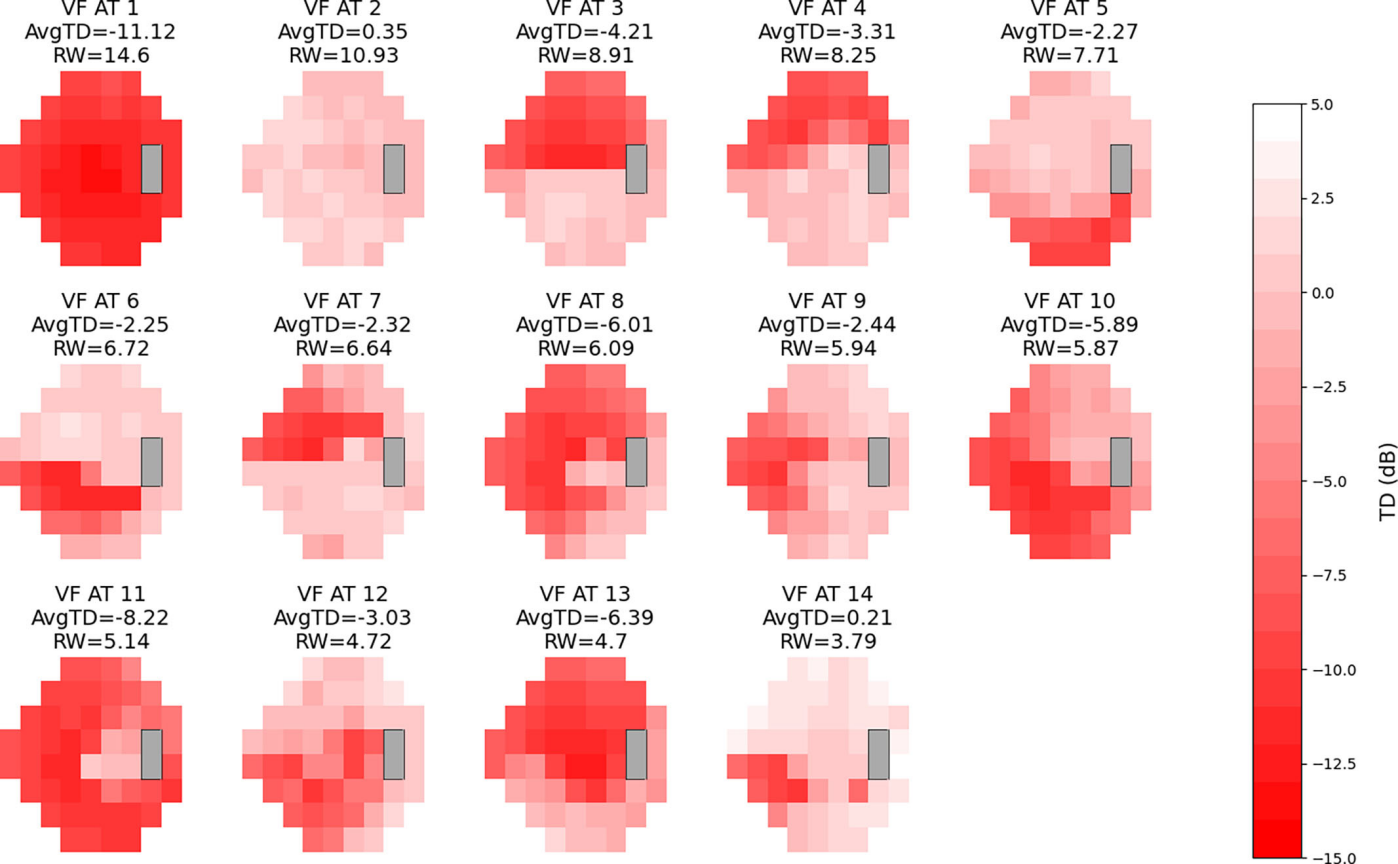
Combined stimulus glaucoma AT map. Combined VF patterns in glaucoma using both stimulus size III and V. The different shades of red within each AT represent TD values, with the scale on the right indicating the corresponding values for each shade. The color scales range from −15 dB to 5 dB. Each AT is displayed with its average TD value and RW as a percentage within the combined dataset. ATs are numbered and presented in order of RW, mirroring integration of patterns from both stimuli.

**Figure 5.**
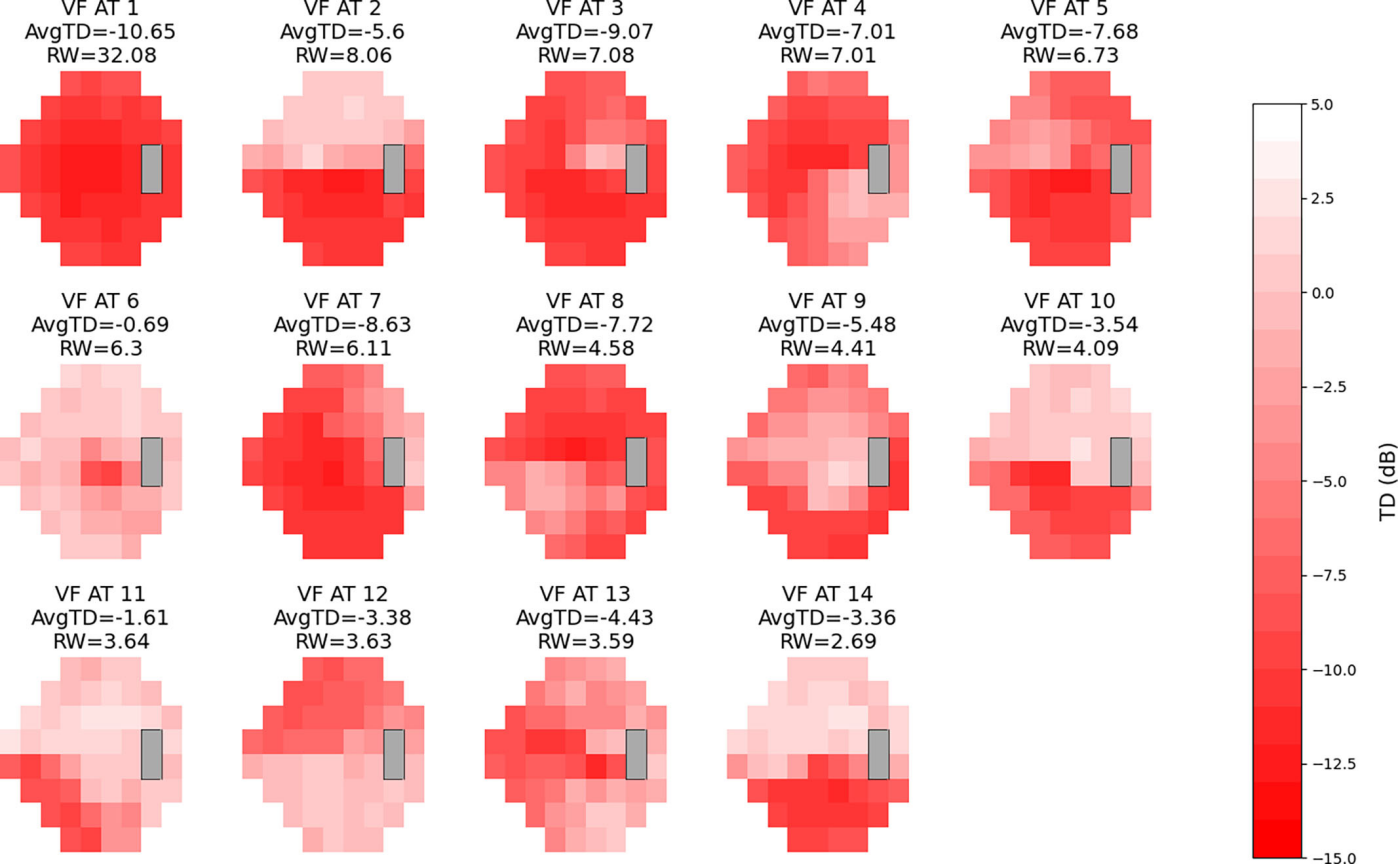
Combined stimulus NAION AT map. Combined VF patterns in NAION using both stimulus size III and V. The different shades of red within each AT represent TD values, with the scale on the right indicating the corresponding values for each shade. The color scales range from −15 dB to 5 dB. Each AT is displayed with its average TD value and RW as a percentage within the combined dataset. ATs are numbered and presented in order of RW, mirroring integration of patterns from both stimuli.

Based on the RSS curves, we chose 14 ATs for both cohorts as it represented a local minimum and was near the global minimum, while still keeping the AT number relatively low (Fig. 1).^9^^–^^11^ We created AT maps for both conditions for the stimulus used (Supplementary Figs. S1–S4). The ATs for each of the stimuli for each condition retained similar patterns and high cosine similarities, with 12 of 14 AT pairings in both conditions achieving a cosine similarity score of 0.8 or greater. Notable exceptions included AT12 (size III) and AT5 (size V) in glaucoma and AT14 (size III) and AT 9 (size V) in NAION (Figs. 2–3; Table 2). These ATs tended to have a low RWs (<8% of the dataset). Combining size III and size V by each condition also resulted in AT maps that were similar to the AT maps for both stimuli respectively (Figs. 4–5). Censored ATs also had similar patterns to an uncensored AA model (Supplementary Fig. S5).

**Table 2.** Cosine Similarity Scores Between Glaucoma and NAION ATs

Glaucoma AT Pairs	Similarity	NAION AT Pairs	Similarity
1, 1	1.00	1, 1	1.00
2, 7	0.99	2, 2	0.99
3, 4	0.96	3, 3	0.99
4, 10	0.67	4, 7	0.97
5, 6	0.96	5, 6	0.92
6, 3	0.95	6, 8	0.97
7, 2	0.96	7, 10	0.98
8, 8	0.98	8, 11	0.83
9, 9	0.92	9, 5	0.98
10, 14	0.97	10, 12	0.93
11, 11	0.95	11, 13	0.97
12, 5	−0.75	12, 14	0.37
13, 12	0.93	13, 4	0.81
14, 13	0.92	14, 9	0.34

Pairs of ATs (size III, size V) derived from archetypal VF data for glaucoma and NAION, displaying the cosine similarity scores between each pair. Most ATs show high similarity scores, indicating strong agreement between the VF patterns for both diseases.

For each disease, respectively, decomposing VFs by stimulus using the combined size III/size V AT map resulted in RWs that were similar for each AT, with an average RW difference of 1.3% for glaucoma and 1.6% for NAION (Supplementary Tables S1–S2). The greatest RW difference was 4.2% for AT2 (superior altitudinal pattern) in glaucoma and 5.0% for AT1 (global severe loss) in NAION. Although overall the RWs are close in value, a Wilcoxon signed-rank test showed significance for differences of most ATs in both conditions (Supplementary Tables S1–S2). However, the distribution appears similar even for significantly different ATs, such as combined glaucoma AT1 (Supplementary Fig. S6).

## Discussion

Our study demonstrates that the VF AT patterns are remarkably similar between stimulus sizes III and V, with AA enabling precise quantification of VF loss patterns in both glaucoma and NAION. The retention of archetypal patterns between size III and size V across both conditions underscores the ability of AA to capture underlying disease characteristics consistently. Our analysis included our recently reported method that optimizes the threshold for censoring that allows equating the stimuli in both glaucoma and NAION.^5^ This censoring threshold seems to be similar for both disorders. Censoring is essential for accurate analysis because data points measured below a particular range are contaminated by noise, rendering them unreliable and unrepeatable.^4^^,^^13^ A high retest variability distorts statistical measures and obscures meaningful patterns.

Despite the large number of NAION TDs being censored, size III effectively captured the same patterns observed with size V. This finding is noteworthy, considering that size V is generally recognized for its superior reliability at low TD values. The fact that size III VFs reproduced archetypal patterns similar to those for size V suggests that AA is resilient to the effects of censored data and can reliably reflect the underlying disease characteristics even with less precise data from size III. It also shows that pattern information is derived from the upper half of the VF dynamic range.

One advantage of using cosine similarity in our analysis is that it ignores VF intensity and focuses solely on pattern similarity. Because AA produces patterns as weighted sums (i.e., different intensities of patterns), cosine similarity allows us to focus on the underlying pattern itself. However, there are two potential disadvantages. First, VFs are two-dimensional measurements, and we could expect neighboring points to be similar owing to spatial correlation or slight variation in participant fixation. The one-dimensional nature of cosine similarity does not account for this spatial relationship. However, this factor would affect both stimuli equally, so comparisons across stimuli would still capture this similarity to some extent.

When decomposing VFs using the combined AT maps, the RWs for each AT were similar to the AT maps for size III and size V. However, there were small, statistically significant differences in the RW for most AT across both conditions. This finding indicates that, although the overall patterns are consistent, the quantification of AT contributions may vary slightly between stimuli. This result suggests potential sensitivity differences for stimulus-specific characteristics or variations in data interpretation. However, although the Wilcoxon test is sensitive to ordering and changes, the RW breakdown and distributions of each AT were still similar. Particularly for NAION AT1, although there is an absolute RW difference of 5%, the AT captures so much of the dataset's information that the 5% difference is a relatively small fraction of the dataset.

Our study has several limitations. Many patients with either disease had VFs that contained large areas of severe deficit, leading us to censor a large number of TDs, particularly in the NAION group. This factor could have potentially artifactually elevated TDs with resulting distortion in the AA. Currently, our VF technology using luminance fixed differential light sensitivity perimetry does not have the capability to measure such severe degrees of vision loss consistently. Using size modulation instead may overcome this deficit. We also used a normative database with only 60 subjects tested twice for calculating TD values for size V. Although this sample size provided a useful reference, it may not fully capture the variability in a larger population. We also selected 14 AT because it represented a balance between minimizing noise and capturing meaningful patterns, even though it may not be the mathematically optimal AT count. This choice allowed us to observe consistent and concordant patterns across both size III and size V stimuli, demonstrating that the selected AT number captured relevant VF structures effectively. Selecting the same number of ATs for both diseases also demonstrated that the number of discordant AT pairs was the same. We also emphasize that 14 ATs should not be assumed as universally optimal for all studies. AT selection may depend on specific datasets, and exploring alternative counts may be required depending on context.

This study highlights the robustness of archetypal patterns, even with varying stimuli and measurement constraints, emphasizing the potential of AA as a dependable tool for clinical and research assessments. Future research could explore integrating AA to evaluate VFs using a variety of stimuli to determine whether similar patterns still persist for glaucoma and NAION. Because prior studies have shown that censoring and age standardization of TDs of size III and size V are directly comparable, we can also consider combining mixed datasets consisting of size III and size V values, even if they were not obtained simultaneously for the same patient at the same time.^5^ Ultimately, our findings affirm the strength of AA in identifying consistent VF patterns across different stimuli, paving the way for enhanced diagnostic precision and personalized treatment strategies in glaucoma and NAION.

## Supplementary Material

Supplement 1
