# Supplementary material for: Archetypal Analysis Reveals Consistent Visual Field Patterns for Stimulus Sizes III and V in Glaucoma and NAION
Source: Transl Vis Sci Technol. 2024 Dec 12;13(12):15. doi: 10.1167/tvst.13.12.15 (PMC11645746; doi:10.1167/tvst.13.12.15)
Supplement: Supplement 1 [file tvst-13-12-15_s001.pdf]

Archetype Relative Weights by Stimulus for Combined Glaucoma AA Map

	Glaucoma Size III	Glaucoma Size V
AT1	**16.1**	**13.1**
AT2	**8.8**	**13.0**
AT3	**9.3**	**8.5**
AT4	8.2	8.3
AT5	**7.1**	**8.3**
AT6	**7.3**	**6.2**
AT7	**5.7**	**7.6**
AT8	**6.5**	**5.7**
AT9	**5.6**	**6.3**
AT10	5.6	6.1
AT11	**5.6**	**4.7**
AT12	**5.2**	**4.2**
AT13	**5.2**	**4.2**
AT14	3.6	3.9

Supplemental table 1. Relative weights of all archetypes by stimulus size for glaucoma. Bold text indicates a significant difference between the two stimuli, as

determined by a Wilcoxon signed-rank test. Although most archetypes display significant differences, the average absolute change between the two stimuli is 1.3%

Archetype Relative Weights by Stimulus for Combined NAION AA Map

	NAION Size III RW	NAION Size V RW
AT1	**34.5**	**29.5**
AT2	**7.2**	**9.0**
AT3	6.8	7.4
AT4	**7.3**	**6.7**
AT5	6.7	6.9
AT6	**8.5**	**4.0**
AT7	**4.2**	**8.0**
AT8	**4.1**	**5.0**
AT9	**4.0**	**4.7**
AT10	**4.8**	**3.4**
AT11	**2.6**	**4.6**
AT12	3.6	3.7
AT13	3.6	3.7
AT14	**2.1**	**3.3**

Supplemental table 2. Relative weights of all archetypes by stimulus size for glaucoma. Bold text indicates a significant difference between the two stimuli, as

determined by a Wilcoxon signed-rank test. Although most archetypes display significant differences, the average absolute change between the two stimuli is 1.6%.

Stimulus Size III Glaucoma Archetype Map

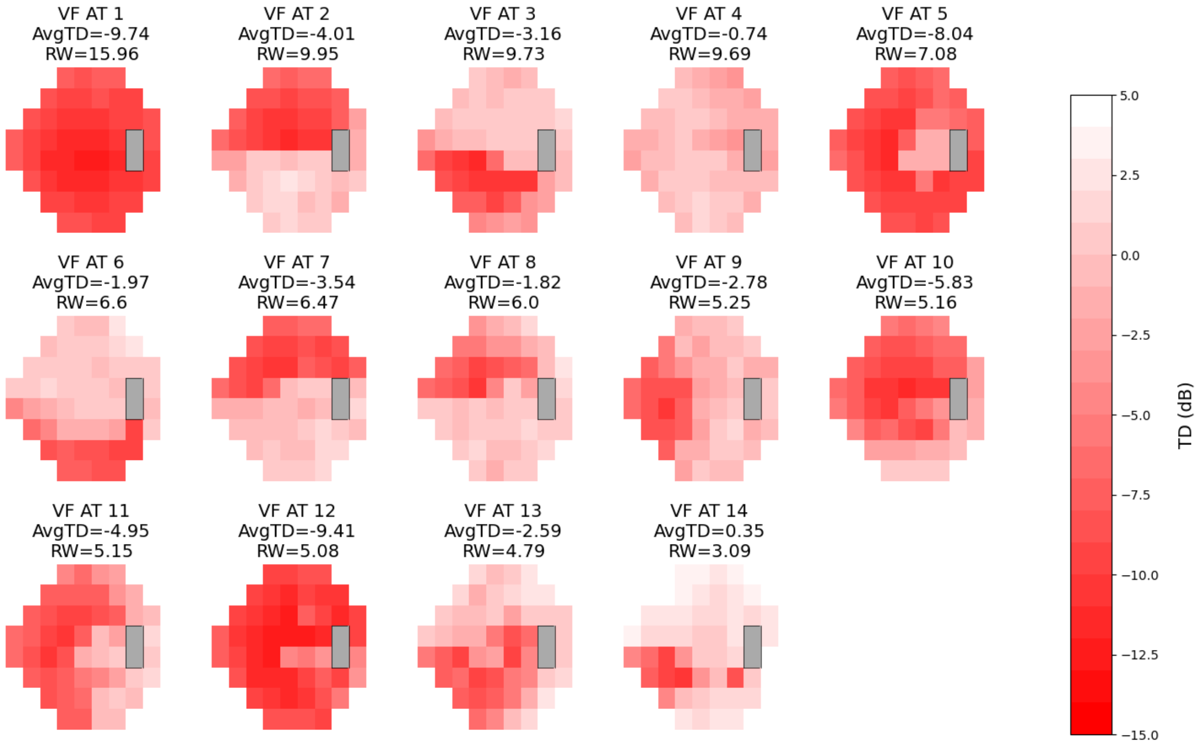

Supplemental Figure 1. Visual field patterns in glaucoma using stimulus size III. The different shades of red within each archetype represent total deviation values, with the scale on the right indicating the corresponding values for each shade. The color scales range from -15 dB to 5 dB. Each archetype is shown along with its corresponding average total deviation value and relative weight as a percentage within the dataset. Archetypes are numbered and presented in order of relative weight.

Stimulus Size V Glaucoma Archetype Map

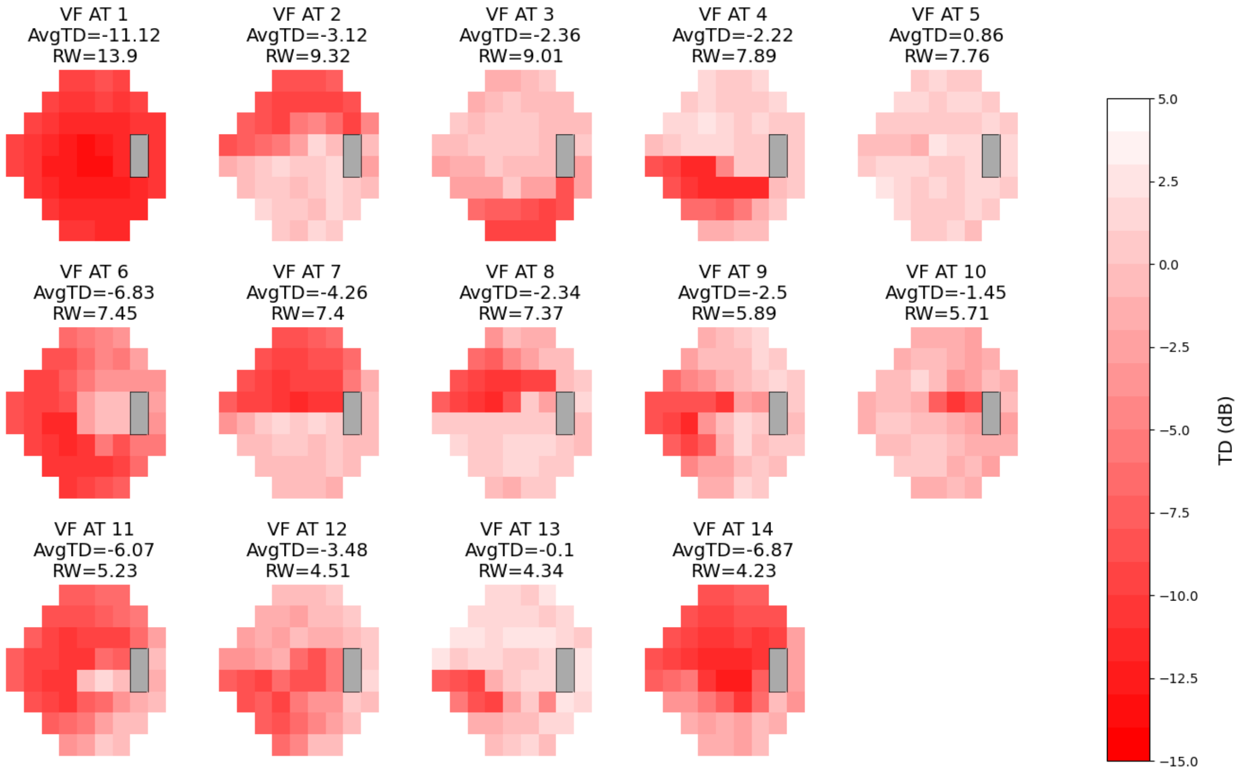

Supplemental Figure 2. Visual field patterns in glaucoma using stimulus size V. The different shades of red within each archetype represent total deviation values, with the scale on the right indicating the corresponding values for each shade. The color scales range from -15 dB to 5 dB. Each archetype is shown along with its corresponding average total deviation value and relative weight as a percentage within the dataset. Archetypes are numbered and presented in order of relative weight.

Stimulus Size III NAION Archetype Map

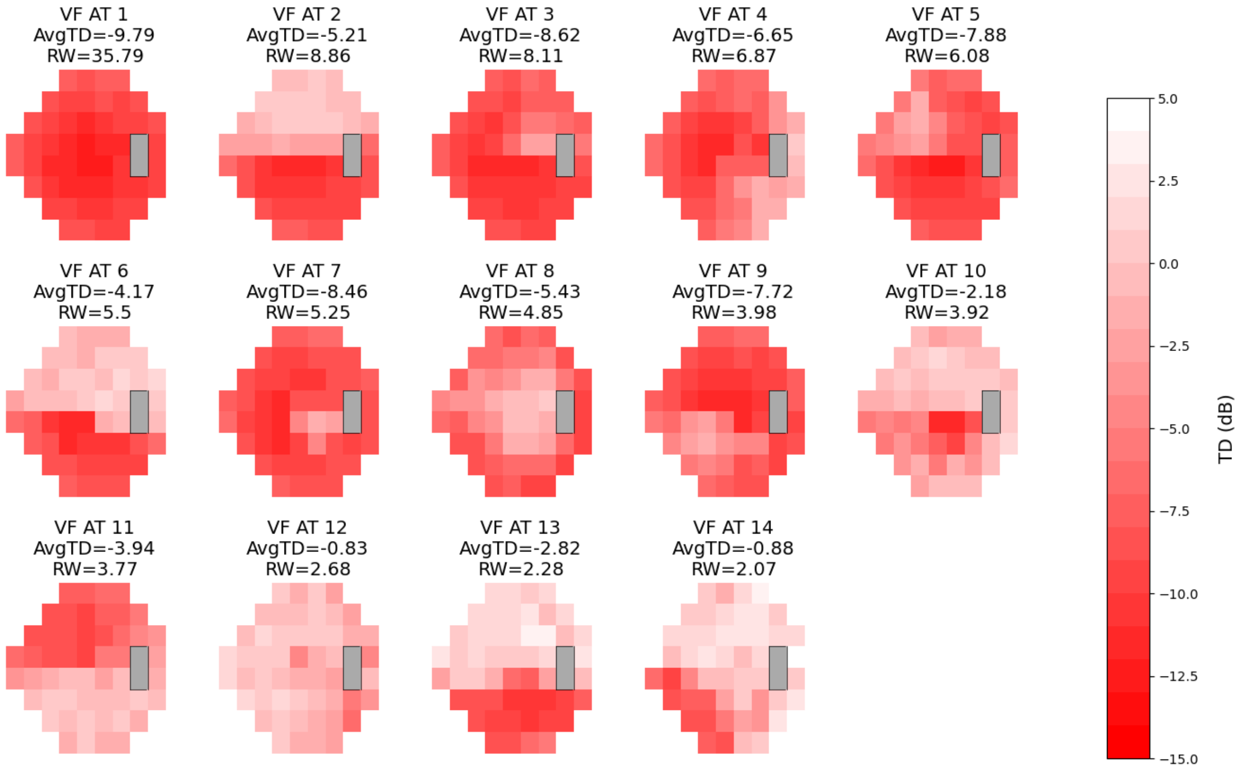

Supplemental Figure 3. Visual field patterns in non-arteritic anterior ischemic optic neuropathy using stimulus size III. The different shades of red within each archetype represent total deviation values, with the scale on the right indicating the corresponding values for each shade. The color scales range from -15 dB to 5 dB. Each archetype is shown along with its corresponding average total deviation value and relative weight as a percentage within the dataset. Archetypes are numbered and presented in order of relative weight.

Stimulus Size V NAION Archetype Map

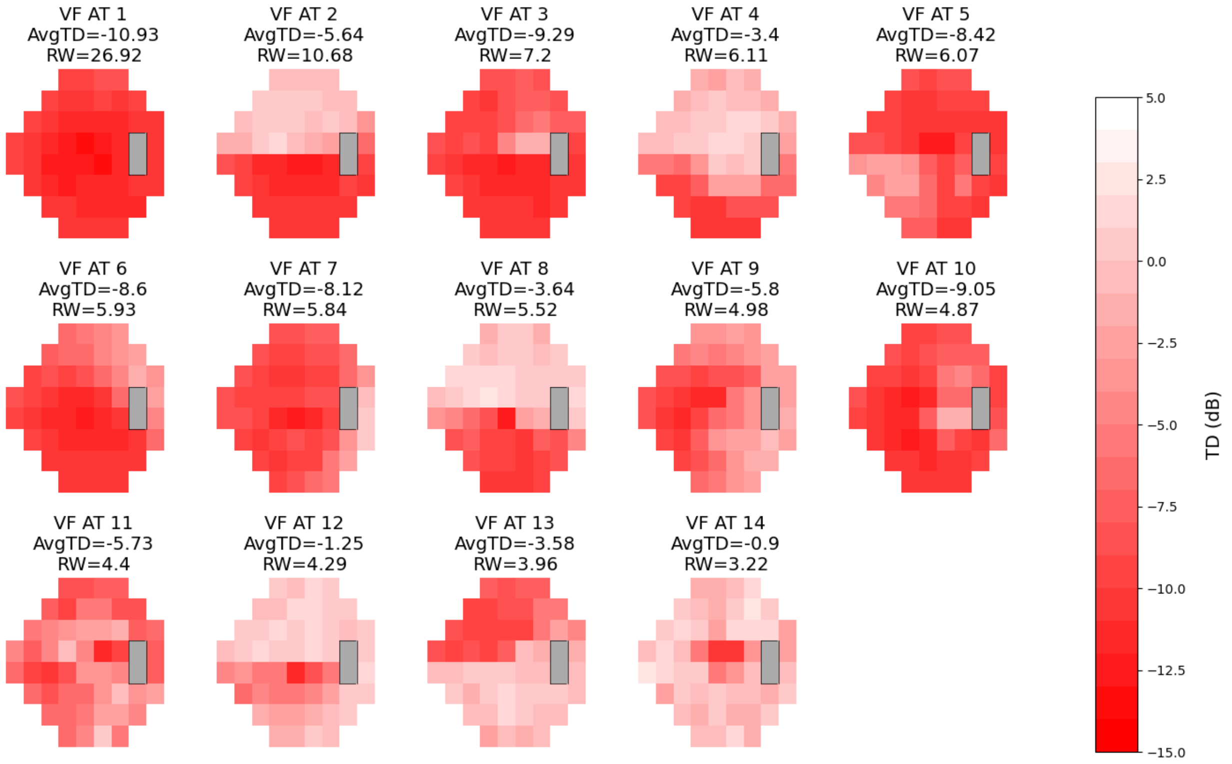

Supplemental Figure 4. Visual field patterns in non-arteritic anterior ischemic optic neuropathy using stimulus size V. The different shades of red within each archetype represent total deviation values, with the scale on the right indicating the corresponding values for each shade. The color scales range from -15 dB to 5 dB. Each archetype is shown along with its corresponding average total deviation value and relative weight as a percentage within the dataset. Archetypes are numbered and presented in order of relative weight.

Uncensored Stimulus Size III NAION Archetype Map

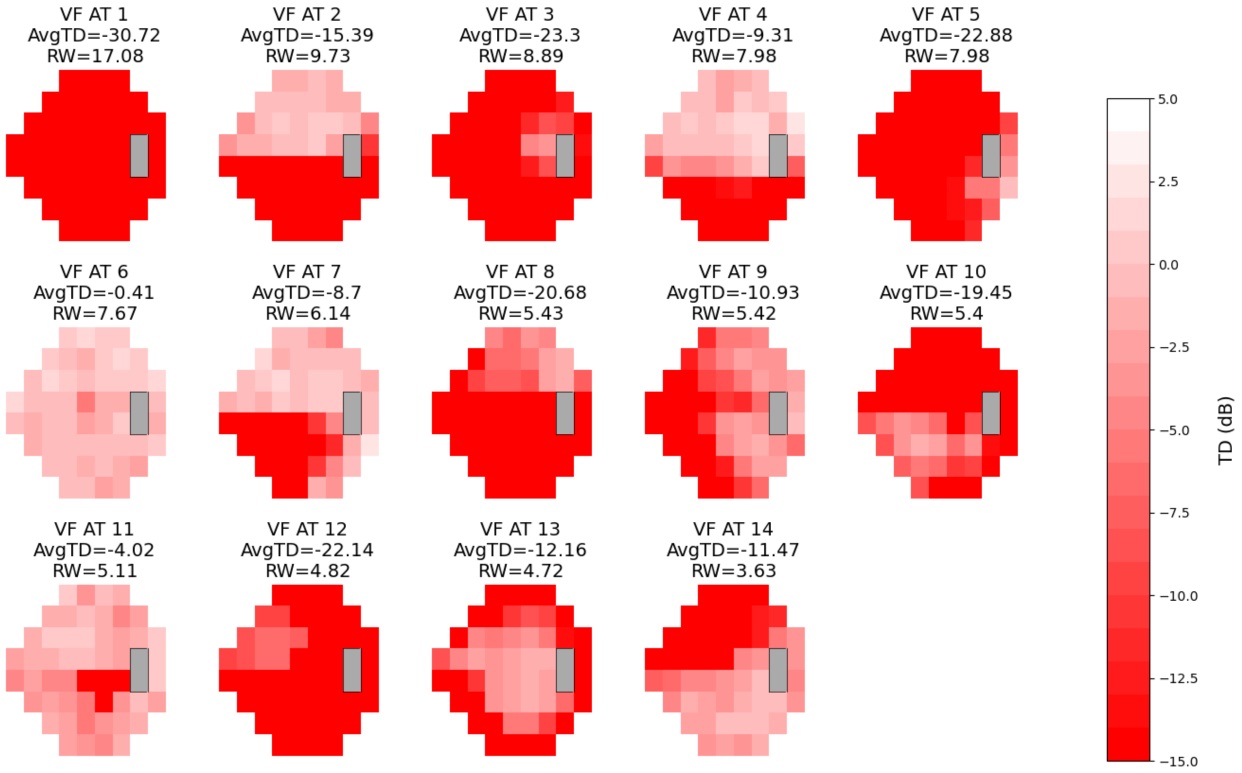

Supplemental Figure 5. Uncensored visual field patterns in non-arteritic anterior ischemic optic neuropathy using stimulus size III. The different shades of red within each archetype represent total deviation values, with the scale on the right indicating the corresponding values for each shade. The color scales range from -15 dB to 5 dB. Each archetype is shown along with its corresponding average total deviation value and relative weight as a percentage within the dataset. Archetypes are numbered and presented in order of relative weight. Patterns are similar to its censored analogue.

Distribution of Glaucoma Visual Fields by Relative Weights of AT1 by Stimulus Type

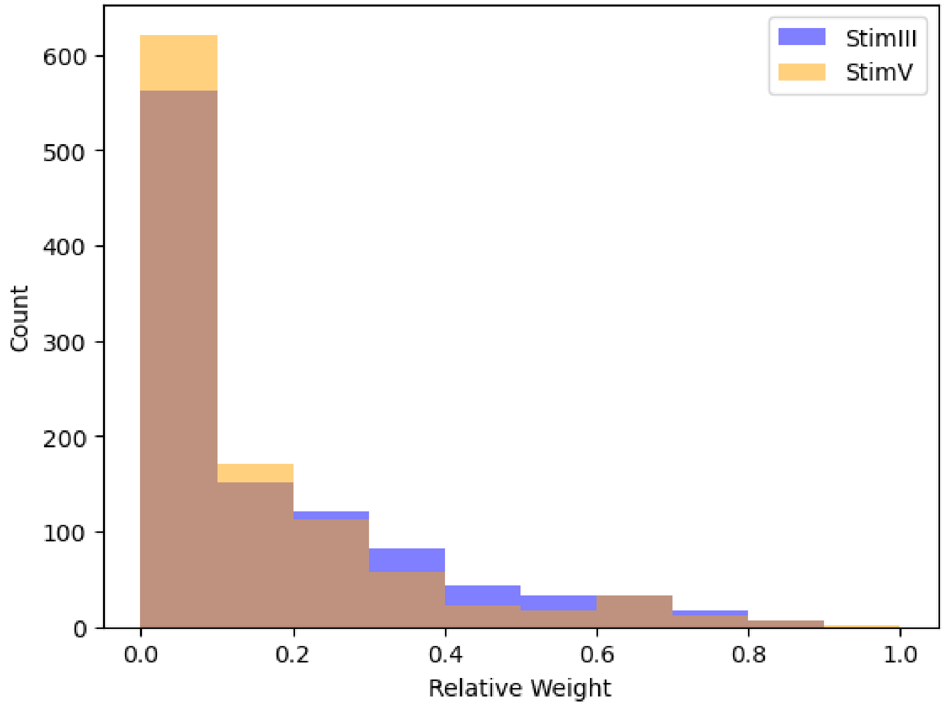

Supplemental Figure 6. Distribution of glaucoma visual fields based on the relative weights of AT1 for the decomposition of the combined glaucoma stimulus model by stimulus type. While there’s a significant difference ($p < 0.001$) in average relative weight (16.1% for stimulus size III vs 13.1% for stimulus size V), the distribution qualitatively looks similar.
